# Not guilty by reason of insanity and dangerousness: A demographic, clinical and forensics description of the patients in the forensic inpatinent service of Coimbra

**DOI:** 10.1192/j.eurpsy.2021.1894

**Published:** 2021-08-13

**Authors:** M. Coroa, D. Pereira, V. Santos, A. Macedo

**Affiliations:** Psychiatry Department, Centro Hospitalar e Universitário de Coimbra, Coimbra, Portugal

## Abstract

**Introduction:**

Despite being essential for the service quality improvement, empirical research on the characteristics of people hospitalized in forensic psychiatry units and the psychopharmacological treatment instituted in this care context is scarce in Portugal.

**Objectives:**

To describe the sociodemographic, clinical and criminological characteristics of the patients admitted to a forensic psychiatric unit in Portugal, as well as, the psychiatric drugs prescription pattern in this care context.

**Methods:**

A retrospective observational study was carried out, through the data analysis of patients admitted to the Sobral Cid Forensic Psychiatry unit of the Coimbra Hospital and University over the past 12 years.

**Results:**

The sample had 194 inpatients, 153(78.9%) male and 41(21.1%) females. The mean age was 43.3 years and 74.7% had no professional, school or occupational activity. The most frequent psychiatric diagnoses were psychotic disorders (56.7%) and neurodevelopmental disorders(33.5%). 24.2% had at least two psychiatric diagnoses and 38.7% had concomitant medical conditions. 77.8% had history of psychiatric hospitalizations and 21.6% had history of self-injurious behaviors. 37.1% of the sample had a criminal record. Crimes against people were the most frequent. The use of injectable antipsychotic formulations was frequent and 18.6% of the patients were medicated with Clozapine. The prescribed daily doses were above the defined daily dose. Psychotic disorders and addictive disorders were less frequent in women. Statistically significant differences were found in the frequency of homicide between females(41.5%) and males(22.2%).

**Conclusions:**

Tailored solutions are crucial to accomplish the purpose of security measures, mostly by addressing the identified needs and rethinking the approach on this specific context.

**Disclosure:**

No significant relationships.

**Keyword:**

Forensic Psychiatry Units

